# Four-Objective Optimizations of a Single Resonance Energy Selective Electron Refrigerator

**DOI:** 10.3390/e24101445

**Published:** 2022-10-11

**Authors:** Jinhu He, Lingen Chen, Yanlin Ge, Shuangshuang Shi, Fang Li

**Affiliations:** 1 Institute of Thermal Science and Power Engineering, Wuhan Institute of Technology, Wuhan 430205, China; 2 Hubei Provincial Engineering Technology Research Center of Green Chemical Equipment, Wuhan 430205, China; 3 School of Mechanical & Electrical Engineering, Wuhan Institute of Technology, Wuhan 430205, China

**Keywords:** energy selective electron, coefficient of performance, cooling load, ecological function, figure of merit, finite time thermodynamics

## Abstract

According to the established model of a single resonance energy selective electron refrigerator with heat leakage in the previous literature, this paper performs multi-objective optimization with finite-time thermodynamic theory and NSGA-II algorithm. Cooling load ($\bar{R}$), coefficient of performance (ε), ecological function ($\bar{ECO}$), and figure of merit ($\bar{\chi }$) of the ESER are taken as objective functions. Energy boundary $\left(E^{\prime }/k_{B}\right)$ and resonance width $\left(\Delta E/k_{B}\right)$ are regarded as optimization variables and their optimal intervals are obtained. The optimal solutions of quadru-, tri-, bi-, and single-objective optimizations are obtained by selecting the minimum deviation indices with three approaches of TOPSIS, LINMAP, and Shannon Entropy; the smaller the value of deviation index, the better the result. The results show that values of $E^{\prime }/k_{B}$ and $\Delta E/k_{B}$ are closely related to the values of the four optimization objectives; selecting the appropriate values of the system can design the system for optimal performance. The deviation indices are 0.0812 with LINMAP and TOPSIS approaches for four-objective optimization ($\bar{ECO}-\bar{R}-\varepsilon -\bar{\chi }$), while the deviation indices are 0.1085, 0.8455, 0.1865, and 0.1780 for four single-objective optimizations of maximum $\bar{ECO}$, $\bar{R}$, ε, and $\bar{\chi }$, respectively. Compared with single-objective optimization, four-objective optimization can better take different optimization objectives into account by choosing appropriate decision-making approaches. The optimal values of $E^{\prime }/k_{B}$ and $\Delta E/k_{B}$ range mainly from 12 to 13, and 1.5 to 2.5, respectively, for the four-objective optimization.

## 1. Introduction

With the development of micro–nano technology, the micro energy conversion systems have gradually entered the field of view of modern thermodynamic researchers. As with motors in the macro world, there are also micro “motors” at the micro level, including quantum thermal cycles [1,2], Brownian motors [3,4], and electron engine systems [5,6,7], etc. They also function as motors in a mechanical sense; that is, they obtain mechanical energy by converting other forms of energy. In the studies on micro energy conversion systems, thermodynamic performance of the system has always been one of the core issues that people pay attention to. Due to their advantages of high efficiency, controllability, and easy integration, the optimal performance of these micro energy conversion systems has become a very active focus of modern thermodynamic research.

Since its establishment, the theory of finite time thermodynamics (FTT) [8,9,10,11,12,13,14,15,16,17,18,19] has been applied to research the optimal configurations [20,21,22,23,24,25,26,27,28,29,30,31,32,33,34,35,36,37,38,39,40,41,42,43] and the optimal performances [44,45,46,47,48,49,50,51,52,53,54,55,56,57,58,59,60,61,62,63,64,65,66,67,68,69,70,71,72,73,74,75,76,77] of thermal devices. The ideas of FTT considering performance optimization with heat leakage loss and finite rate heat transfer are also applicable to analyses and optimizations of micro energy conversion systems. Applying FTT to optimize micro energy conversion systems has not only achieved many research results of both theoretical significance and practical application value, but also broadened the application scope of the theory.

The model of an energy selective electron (ESE) heat engine was first established in 2002 [78], its working fluid constitutes electrons that are stored in two electron reservoirs and propagated through an energy filter. Electrons transfer under the influence of deflection voltage, temperature difference, and electrochemical potential difference. Since electrons carry energy, energy transfer also occurs during the transmission process, thereby realizing energy conversion.

In the research process of ESERs, in addition to the two basic performance indicators of cooling load (CL) and coefficient of performance (COP), there are also the figure of merit (FOM) [79,80,81] and ecological function [82,83,84]. Yan [79] first took the product of CL and COP as the optimization goal of the refrigerators in 1984; de Tomas et al. [80] named it as the FOM optimization criterion of the refrigerators and it was introduced into low-dissipation heat engines [81]. Angulo-Brown [82] first proposed the ecological function of heat engines in 1991 and it was defined as $E^{\prime \prime }=P-T_{L}\sigma$, where P represents the power output, $T_{L}$ represents the temperature of the cold reservoir, and σ represents the entropy generation rate. Yan [83] thought that this ecological function did not inherently distinguish heat (energy) from work (exergy), and believed comparing exergy (P) and non-exergy ($T_{L}\sigma$) was incomplete; then, he modified the ecological function to be $E^{\prime }=P-T_{0}\sigma$, where $T_{0}$ represents the environment temperature. Chen et al. [84] further proposed a unified ecological function for various thermodynamic cycles based on exergy and it was defined as $E=A/\tau -T_{0}\sigma$, where A represents the exergy output and τ represents the cycle period. The ecological function and FOM can also take into account the COP and CL of the ESER. Humphrey [85] studied the COP and Cl performances of an endoreversible ESER, and Li et al. [86] studied its ecological function performance. When further considering heat leakage in ESERs, He et al. [87] studied the optimal performance of an irreversible ESER; Ding et al. [88] studied its optimal performance in two operating modes, and Zhou et al. [89] introduced the FOM and ecological function into it.

The performance optimizations mentioned above for ESERs are only aimed at a single optimization objective and there is usually only one optimal solution. With the increase of performance indicators, conflicts will occur among various indicators. In order to take different performance indicators into account and obtain the optimal design scheme, Deb et al. [90] proposed the non-dominated sorting genetic algorithm II (NSGA-II), which overcame the three shortcomings of NSGA, including the high computational complexity of non-dominated sorting, the lack of elite strategies, and the need to specify shared parameters. NSGA-II was widely used for the multi-objective optimization (MOO) of various thermodynamic cycles [91,92,93,94,95,96,97,98,99]. The Brayton cycle [91] and Stirling-Otto combined cycle [92] were optimized by MOO, and the utilized optimization objectives were power output and thermal efficiency; the optimization results were obtained and compared by applying different decision-making approaches. The porous-medium cycle [93] and Dual cycle [94] were optimized by MOO and the utilized optimization objectives were power density, power output, ecological function, and thermal efficiency; in addition, the effects of the compression ratio on four optimization objectives were analyzed. The Organic Rankine cycle [95] was optimized by applying MOO and its performances of pure and mixture working fluids were compared and studied. The ESE heat engine [96] was optimized by MOO and the utilized optimization objectives were ecological function, power output, efficient power, and thermal efficiency. Stirling engines [97,98], the membrane reactor [99], and the bidirectional-ribbed microchannel [100] were also optimized by applying MOO; and the schemes with less contradictions and conflicts were obtained.

There is no work concerning MOO for ESERs in open literature. On the basis of a model of a single resonance ESER with heat leakage established in Ref. [89], this paper will take resonance width and energy boundary as optimization variables to carry out MOO for the ESER with four objectives of CL, COP, FOM, and ecological function by applying the NSGA-II algorithm. The optimal design scheme will be obtained by comparing deviation indices of different optimization objectives combinations with LINMAP, TOPSIS, and Shannon Entropy (SE) approaches.

## 2. Model Description and Performance Indicators

**Figure 1:**
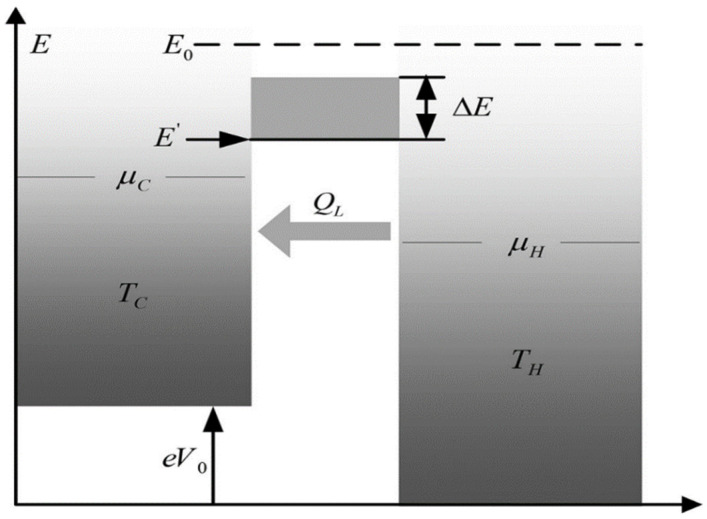
Model of ESER with heat leakage [89].

Figure 1 gives a model of ESER with heat leakage [89]. It includes two electron reservoirs and an energy filter, which only allows electrons in a certain energy range to pass through. In the cold- and hot-electron reservoirs, the electrochemical potentials are $\mu_{C}$ and $\mu_{H}$, and the temperatures are $T_{C}$ and $T_{H}$, respectively. ΔE and $E^{\prime }$ are important structural parameters of the energy filter, and they are the energy boundary and resonance width, respectively. According to Ref. [78], to make the ESE system work as an ESER, the value of the energy E of electrons involved in energy transfer must meet $\mu_{C}<E<E_{0}$, where $E_{0}=(\mu_{C}T_{H}-\mu_{H}T_{C})/(T_{H}-T_{C})$. During the working of the ESER, the heat transfer of electrons is irreversible due to the heat leakage loss caused by phonon propagation.

According to Refs. [78,85], in a small energy range, the heat transferred into hot-reservoir and from cold-reservoir are:

(1) $${\dot{q}}_{H}=\frac{2}{h}(E-\mu_{H})(f_{C}-f_{H})\delta E$$

(2) $${\dot{q}}_{C}=\frac{2}{h}(E-\mu_{C})(f_{C}-f_{H})\delta E$$

where h is Planck constant, and $f_{C}$ and $f_{H}$ are Fermi distributions of electrons:

(3) $$f_{C}={\left[1+\exp(\frac{E-\mu_{C}}{k_{B}T_{C}})\right]}^{-1}$$

(4) $$f_{H}={\left[1+\exp(\frac{E-\mu_{H}}{k_{B}T_{H}})\right]}^{-1}$$

where $k_{B}$ is Boltzmann constant.

From Equations (1)–(4), the total heat transferred into hot-reservoir (${\dot{Q}}_{HE}$) and from cold-reservoir (${\dot{Q}}_{CE}$) are

(5) $${\dot{Q}}_{HE}=\frac{2}{h}\int_{E^{\prime }}^{E^{\prime }+\Delta E}(E-\mu_{H})(f_{C}-f_{H})dE$$

(6) $${\dot{Q}}_{CE}=\frac{2}{h}\int_{E^{\prime }}^{E^{\prime }+\Delta E}(E-\mu_{C})(f_{C}-f_{H})dE$$

The heat leakage loss rate (${\dot{Q}}_{L}$) caused by phonon propagation is

(7) $${\dot{Q}}_{L}=k_{L}(T_{H}-T_{C})$$

where $k_{L}$ is heat-leakage coefficient.

Combining Equations (5)–(7), the actual heats transferred into hot-reservoir (${\dot{Q}}_{H}$) and from cold -reservoir (${\dot{Q}}_{C}$) are

(8) $${\dot{Q}}_{H}={\dot{Q}}_{HE}-{\dot{Q}}_{L}$$

(9) $${\dot{Q}}_{C}={\dot{Q}}_{CE}-{\dot{Q}}_{L}$$

During the numerical solution, $R_{H}=\frac{E^{\prime }+\Delta E-\mu_{H}}{k_{B}T_{H}}$, $r_{H}=\frac{E^{\prime }-\mu_{H}}{k_{B}T_{H}}$, $R_{C}=\frac{E^{\prime }+\Delta E-\mu_{C}}{k_{B}T_{C}}$ and $r_{C}=\frac{E^{\prime }-\mu_{C}}{k_{B}T_{C}}$ are set, and Nielsen function g(x)=PolyLog(2,−x) [101] is introduced.

From Equation (9), the CL (R) of the ESER can be obtained by

(10) $$\begin{array}{ll} R & ={\dot{Q}}_{C}\\ & =\frac{2}{h}(k_{B}T_{C}{)}^{2}[\frac{1}{2}R_{C}^{2}-g(e^{\left(R_{C}\right)})-R_{C}\ln(1+e^{\left(R_{C}\right)})-\frac{1}{2}r_{C}^{2}+g(e^{\left(r_{C}\right)})+r_{c}\ln(1+e^{\left(r_{C}\right)})]\\ & -\frac{2}{h}(k_{B}T_{H}{)}^{2}[\frac{1}{2}R_{H}^{2}-\frac{1}{2}r_{H}^{2}-R_{H}\ln(1+e^{\left(R_{H}\right)})+r_{H}\ln(1+e^{\left(r_{H}\right)})-g(e^{\left(R_{H}\right)})\\ & +g(e^{\left(r_{H}\right)})]+\frac{2}{h}(k_{B}T_{H})(\mu_{C}-\mu_{H})[\ln\frac{\left(1+e^{\left(r_{H}\right)}\right)}{\left(1+e^{\left(R_{H}\right)}\right)}+R_{H}-r_{H}]-k_{L}(T_{H}-T_{C}) \end{array}$$

From Equations (8)–(10), the COP (ε) can be expressed as

(11) $$\begin{array}{cc} \varepsilon & =\frac{R}{{\dot{Q}}_{H}-{\dot{Q}}_{C}}\\ & =\{\frac{2}{h}{\left(k_{B}T_{C}\right)}^{2}\left[\frac{1}{2}R_{C}^{2}+r_{C}\ln\left(1+e^{\left(r_{C}\right)}\right)+g\left(e^{\left(r_{C}\right)}\right)-R_{C}\ln\left(1+e^{\left(R_{C}\right)}\right)-g\left(e^{\left(R_{C}\right)}\right)-\frac{1}{2}r_{C}^{2}\right]\\ & -\frac{2}{h}{\left(k_{B}T_{H}\right)}^{2}\left[\frac{1}{2}R_{H}^{2}+r_{H}\ln\left(1+e^{\left(r_{H}\right)}\right)+g\left(e^{\left(r_{H}\right)}\right)-\frac{1}{2}r_{H}^{2}-R_{H}\ln\left(1+e^{\left(R_{H}\right)}\right)-g\left(e^{\left(R_{H}\right)}\right)\right]\\ & +\frac{2}{h}k_{B}T_{H}\left(\mu_{C}-\mu_{H}\right)\left[R_{H}+\ln\frac{\left(1+e^{\left(r_{H}\right)}\right)}{\left(1+e^{\left(R_{H}\right)}\right)}-r_{H}\right]-k_{L}\left(T_{H}-T_{C}\right)\}\\ & /\left\{\frac{2}{h}k_{B}\left[T_{H}\ln\frac{1+e^{\left(r_{H}\right)}}{1+e^{\left(R_{H}\right)}}-T_{C}\ln\frac{1+e^{\left(r_{C}\right)}}{1+e^{\left(R_{C}\right)}}\right]\left(\mu_{H}-\mu_{C}\right)\right\} \end{array}$$

Combining Equations (10) and (11), the FOM (χ) [79,80] can be given by

(12) $$\begin{array}{cc} \chi & =R\times \varepsilon \\ & =\{\frac{2}{h}{\left(k_{B}T_{C}\right)}^{2}\left[\frac{1}{2}R_{C}^{2}+r_{C}\ln\left(1+e^{\left(r_{C}\right)}\right)+g\left(e^{\left(r_{C}\right)}\right)-R_{C}\ln\left(1+e^{\left(R_{C}\right)}\right)-g\left(e^{\left(R_{C}\right)}\right)-\frac{1}{2}r_{C}^{2}\right]\\ & -\frac{2}{h}{\left(k_{B}T_{H}\right)}^{2}\left[\frac{1}{2}R_{H}^{2}+r_{H}\ln\left(1+e^{\left(r_{H}\right)}\right)+g\left(e^{\left(r_{H}\right)}\right)-\frac{1}{2}r_{H}^{2}-R_{H}\ln\left(1+e^{\left(R_{H}\right)}\right)-g\left(e^{\left(R_{H}\right)}\right)\right]\\ & +\frac{2}{h}k_{B}T_{H}\left(\mu_{C}-\mu_{H}\right)\left[R_{H}-r_{H}+\ln\frac{\left(1+e^{\left(r_{H}\right)}\right)}{\left(1+e^{\left(R_{H}\right)}\right)}\right]-k_{L}\left(T_{H}-T_{C}\right){\}}^{2}\\ & /\left\{\frac{2}{h}k_{B}\left[T_{H}\ln\frac{1+e^{\left(r_{H}\right)}}{1+e^{\left(R_{H}\right)}}-T_{C}\ln\frac{1+e^{\left(r_{C}\right)}}{1+e^{\left(R_{C}\right)}}\right]\left(\mu_{H}-\mu_{C}\right)\right\} \end{array}$$

For the ESER, entropy generation rate (σ) and exergy output rate (A) are, respectively

(13) $$\sigma ={\dot{Q}}_{H}/T_{H}-{\dot{Q}}_{C}/T_{C}$$

(14) $$A={\dot{Q}}_{C}(T_{0}/T_{C}-1)-{\dot{Q}}_{H}(T_{0}/T_{H}-1)$$

Combining Equations (13) and (14), and based on the definition formula of ecological function $ECO=A-T_{0}\sigma$ [84], one has

(15) $$\begin{array}{cc} ECO & =\frac{2}{h}k_{B}\left(\mu_{H}-\mu_{C}\right)\left[T_{H}\ln\frac{1+e^{\left(r_{H}\right)}}{1+e^{\left(R_{H}\right)}}-T_{C}\ln\frac{1+e^{\left(r_{C}\right)}}{1+e^{\left(R_{C}\right)}}\right]-2T_{0}\{\frac{2}{h}k_{B}^{2}\frac{T_{C}\left(T_{H}-T_{C}\right)}{T_{H}}\times \\ & \left[g\left(e^{\left(r_{C}\right)}\right)-g\left(e^{\left(R_{C}\right)}\right)+\frac{1}{2}R_{C}^{2}+r_{C}\ln\left(1+e^{\left(r_{C}\right)}\right)-\frac{1}{2}r_{C}^{2}-R_{C}\ln\left(1+e^{\left(R_{C}\right)}\right)\right]-\frac{2}{h}k_{B}^{2}\times \\ & \frac{T_{H}\left(T_{H}-T_{C}\right)}{T_{C}}\left[\frac{1}{2}R_{H}^{2}+g\left(e^{\left(r_{H}\right)}\right)+r_{H}\ln\left(1+e^{\left(r_{H}\right)}\right)-g\left(e^{\left(R_{H}\right)}\right)-R_{H}\ln\left(1+e^{\left(R_{H}\right)}\right)-\frac{1}{2}r_{H}^{2}\right]\\ & +\frac{2}{h}k_{B}\left(\mu_{C}-\mu_{H}\right)\left[\left(R_{C}-r_{C}\right)\left(1-\frac{T_{C}}{T_{H}}\right)+\frac{T_{H}}{T_{C}}\ln\frac{1+e^{\left(r_{H}\right)}}{1+e^{\left(R_{H}\right)}}-\frac{T_{C}}{T_{H}}\ln\frac{1+e^{\left(r_{C}\right)}}{1+e^{\left(R_{C}\right)}}\right]+{\left(T_{H}-T_{C}\right)}^{2}\frac{k_{L}}{T_{H}T_{C}}\} \end{array}$$

The dimensionless CL (R), FOM (χ), and ecological function ($\bar{ECO}$) are defined as:

(16) $$\bar{R}=R/R_{\max}$$

(17) $$\bar{\chi }=\chi /\chi_{\max}$$

(18) $$\bar{ECO}=ECO/ECO_{\max}$$

## 3. Multi-Objective Optimizations

**Figure 2:**
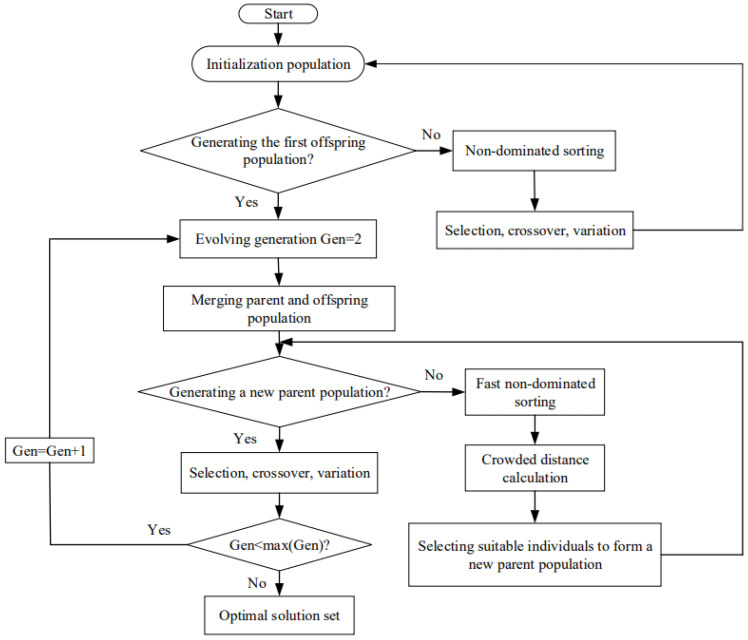
NSGA-II algorithm.

In the MOO process, the solution set is not the only one, but a series of feasible alternatives; that is, the optimal solutions set in the multivariate space, which are called the “Pareto frontiers”. They are obtained by NSGA-II (see Figure 2 for flow chart). The steps of NSGA-II are: (1) the initialization population with size *N* is performed by non-dominated sorting; then, the first offspring population is obtained by selection, crossover, and variation through genetic algorithm; (2) in the second generation, merging the parent population and first offspring population for rapid non-dominated sorting, the crowding distance of individuals is calculated to select suitable individuals to form a new parent population; and (3) a new population of offspring is obtained with the genetic algorithm; when it meets the program requirements, the genetic algorithm will end and a Pareto optimal frontier will be obtained.

NSGA-II is an improvement for the shortcomings of NSGA, which can improve the convergence speed of the algorithm and maintain the diversity of results by adopting fast hierarchical sorting and elite strategies. In order to obtain an ideal design scheme by applying NSGA-II, three strategies including TOPSIS, LINMAP, and SE are usually adopted and their pros and cons are compared by deviation indices (Ds) obtained from them. The positive and negative ideal points are the solutions at which all performance indicators of Pareto frontiers reach the maximum and minimum values, respectively. TOPSIS strategy makes the solutions farthest from the negative ideal point, LINMAP strategy makes the solutions closest to positive ideal points, and SE strategy attains the solutions when the last performance indicator reaches its maximum.

The D represents the average distance between the solution and positive ideal point; the smaller the value, the better the result. The definition of D is

(19) $$D=\frac{\sqrt{\sum_{j=1}^{m}{\left(G_{j}-G_{j}^{\mathrm{positive}}\right)}^{2}}}{\sqrt{\sum_{j=1}^{m}{\left(G_{j}-G_{j}^{\mathrm{positive}}\right)}^{2}}+\sqrt{\sum_{j=1}^{m}{\left(G_{j}-G_{j}^{\mathrm{negative}}\right)}^{2}}}$$

where $G_{j}$ is value of the *j*-th goal, $G_{j}^{\mathrm{positive}}$ is value of the *j*-th goal of positive ideal point, and $G_{j}^{\mathrm{negative}}$ is value of the *j*-th goal of negative ideal point.

According to Ref. [89], $k_{L}=1.5\times 10^{-14}\;W/K$, $T_{0}=1.6\;K$, $T_{C}=1.2\;K$, $\mu_{C}/k_{B}=12\;K$, $T_{H}=2.2\;K$ and $\mu_{H}/k_{B}=10\;K$ are set. The value ranges of energy boundary $E^{\prime }/k_{B}$ and resonance width $\Delta E/k_{B}$ are set to 10~15 and 0~5, respectively.

Table 1 lists the setting parameters of NSGA-II. Table 2 gives the outcomes of various single-objective and multi-objective optimizations. From Table 2, the Ds are 0.0812 with LINMAP and TOPSIS approaches when the MOO is performed on four-objective optimization ($\bar{ECO}-\bar{R}-\varepsilon -\bar{\chi }$), while the Ds are 0.1085, 0.8455, 0.1865, and 0.1780 for four single-objective optimizations of maximum $\bar{ECO}$, $\bar{R}$, ε, and $\bar{\chi }$, respectively. It indicates that compared with single-objective optimization, MOO can better take into account different optimization objectives by choosing appropriate decision-making methods. For the MOO of $\bar{ECO}-\bar{R}$, the D obtained with the TOPSIS approach is 0.0809, which is the smallest and the most perfect scheme; and the corresponding values of the $E^{\prime }/k_{B}$ and $\Delta E/k_{B}$ are 12.5887 and 1.8050, respectively.

**Figure 3:**
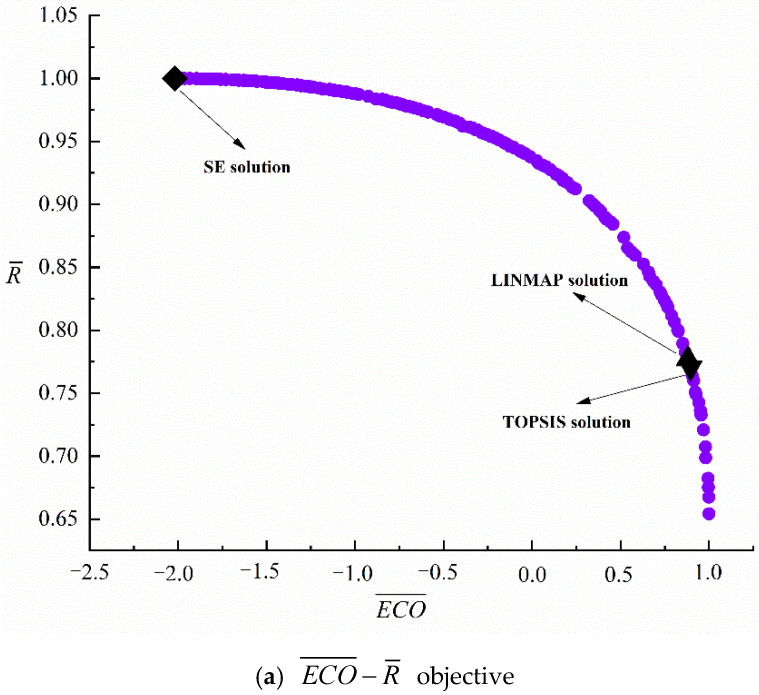

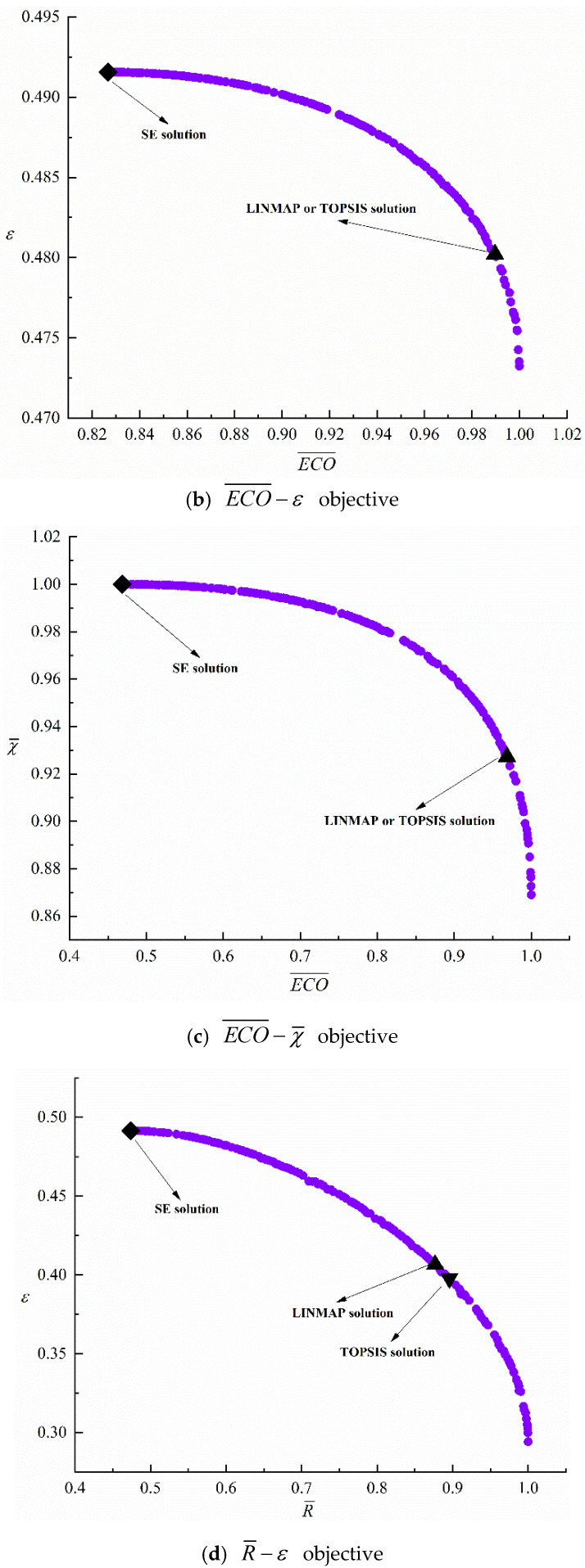

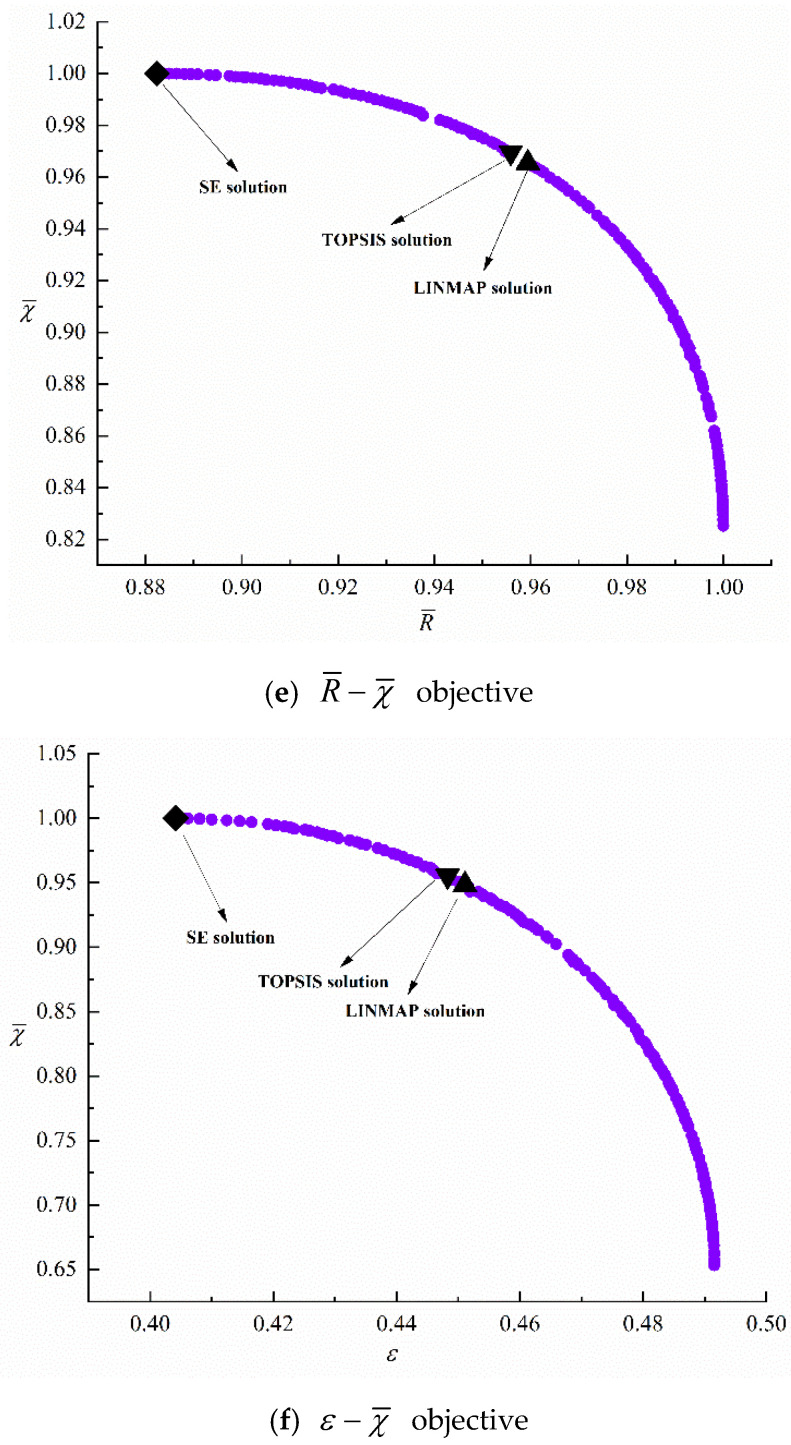
Pareto frontiers for different two-objective optimizations.

Figure 3 gives the outcomes of different (six) bi-objective optimization combinations. It can be concluded from Figure 3 that two different performance indicators cannot achieve the best at the same time, and the improvement of one performance indicator will often lead to the deterioration of the other. From Figure 3 and Table 2, SE solutions represent the points when the performance indicators on the ordinate reach the maximum, LINMAP and TOPSIS solutions represent the points closest to the positive ideal point (1.0000, 1.0000, 0.4916, 1.0000) and the points farthest from the negative ideal point (−2.0171, 0.4739, 0.2943, 0.6532), respectively. As $\bar{ECO}$ grows, $\bar{R}$, ε, and $\bar{\chi }$ all decline. As $\bar{R}$ grows, both ε and $\bar{\chi }$ decline. As ε grows, $\bar{\chi }$ declines. From Table 2, for the MOOs of $\bar{ECO}-\varepsilon$ and $\bar{ECO}-\bar{\chi }$, the Ds (0.1229, 0.0884) obtained with the LINMAP approach are the same as those obtained with the TOPSIS approach, and are better than those obtained with the SE approach. For the MOOs of $\bar{ECO}-\bar{R}$, $\bar{R}-\varepsilon$, and $\varepsilon -\bar{\chi }$, the Ds (0.0809, 0.3786, 0.0814) obtained with the TOPSIS approach are better than those obtained with LINMAP and SE approaches. For the MOO of $\bar{R}-\bar{\chi }$, the D (0.1780) obtained with SE is more perfect than that obtained with LINMAP and TOPSIS approaches.

**Figure 4:**
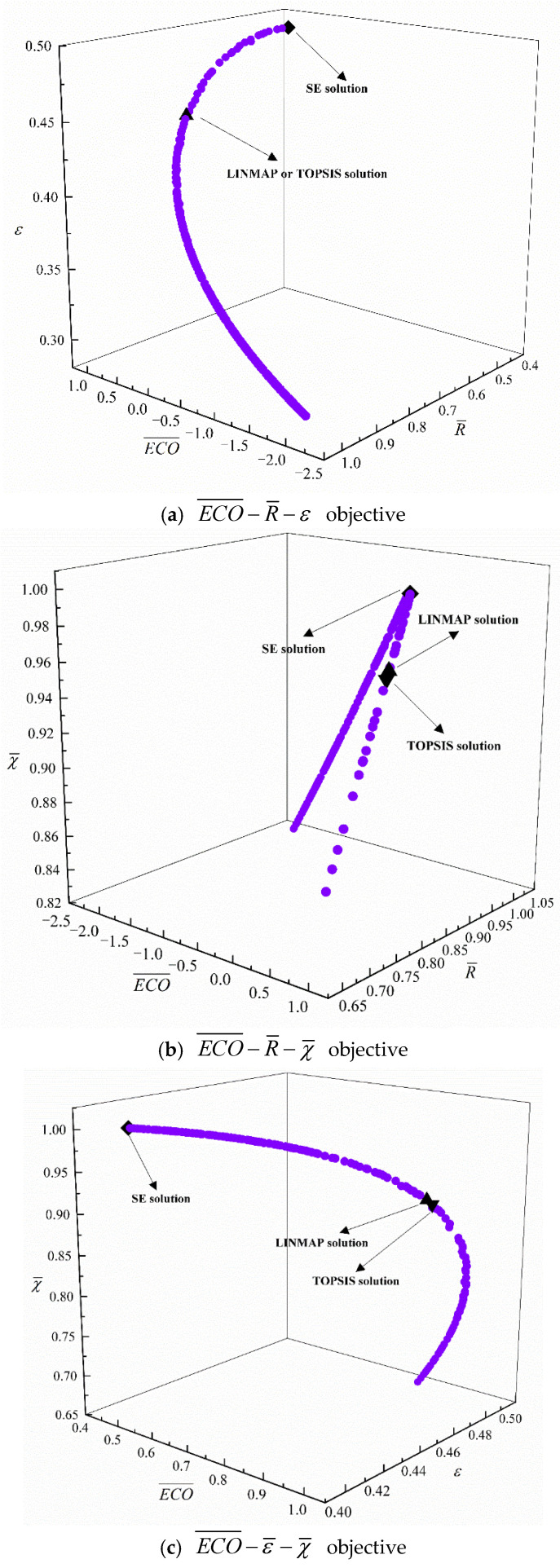

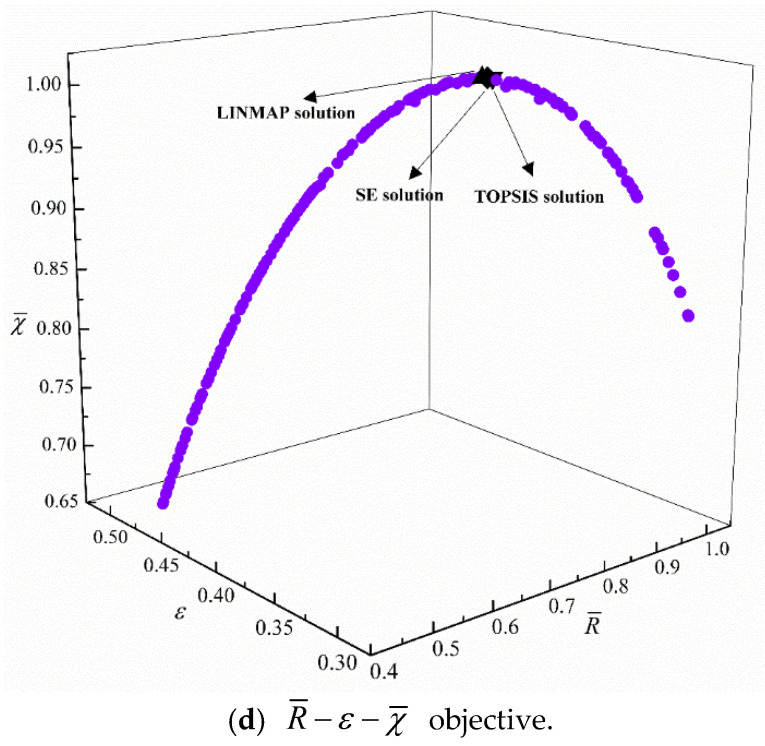
Pareto frontiers for different three-objective optimizations.

Figure 4 gives the outcomes of different (four) tri-objective optimization combinations. As $\bar{ECO}$ grows, $\bar{R}$ declines, ε grows, and $\bar{\chi }$ first grows and then declines. As $\bar{R}$ grows, ε declines, and $\bar{\chi }$ first grows and then declines. From Table 2, for the MOO of $\bar{ECO}-\bar{R}-\varepsilon$, the D (0.0814) obtained with the LINMAP approach is the same as that obtained with the TOPSIS approach, and is better than that obtained with the SE approach. For the MOO of $\bar{ECO}-\bar{R}-\bar{\chi }$, the D (0.0816) obtained with the TOPSIS approach is better than that obtained with LINMAP and SE approaches. For the MOO of $\bar{ECO}-\varepsilon -\bar{\chi }$ and $\bar{R}-\varepsilon -\bar{\chi }$, the Ds (0.0888, 0.1692) obtained with the LINMAP approach are better than those obtained with TOPSIS and SE approaches.

**Figure 5:**
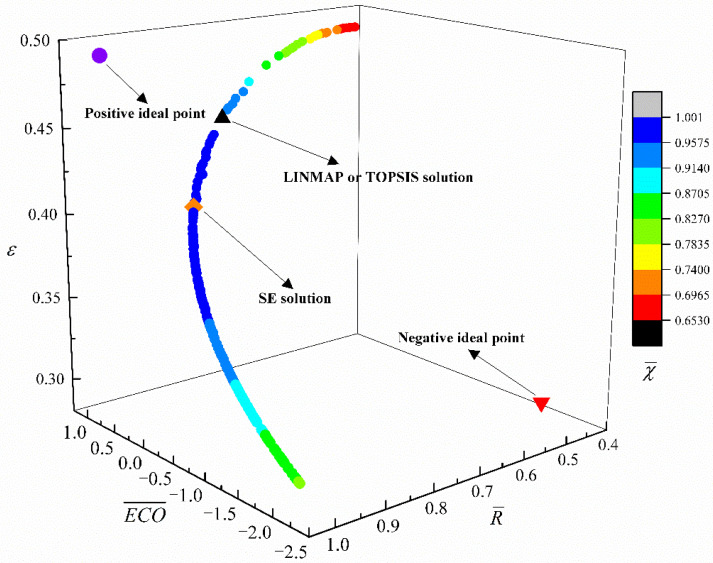
Pareto frontier for $\bar{ECO}-\bar{R}-\varepsilon -\bar{\chi }$ optimization.

Figure 5 gives the Pareto frontier for four-objective ($\bar{ECO}-\bar{R}-\varepsilon -\bar{\chi }$) optimization. The numerical changes of $\bar{R}$ and ε are represented by three axes, and the numerical change of $\bar{\chi }$ is represented by color change. The positive and negative points lie outside the Pareto frontier, which means that the four optimization objectives $\bar{ECO}$, $\bar{R}$, ε, and $\bar{\chi }$ cannot be optimal or worst at the same time. As $\bar{ECO}$ grows, $\bar{R}$ declines, ε grows, and $\bar{\chi }$ first grows and then declines. From Table 2, for the MOO of $\bar{ECO}-\bar{R}-\varepsilon -\bar{\chi }$, the Ds (0.0812, 0.0812) obtained with LINMAP and TOPSIS approaches are equal, which are more reasonable than those obtained with SE.

**Figure 6:**
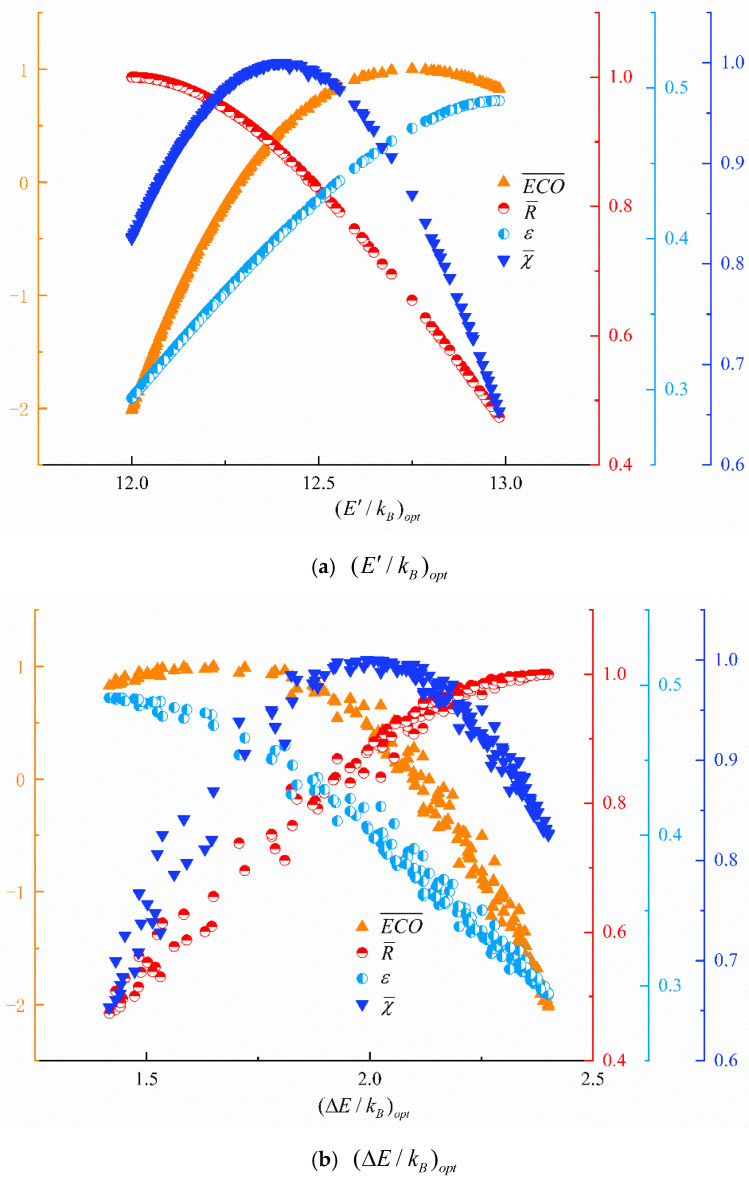
Distributions of design variables in Pareto frontier.

Figure 6 gives the distributions of ${\left(E^{\prime }/k_{B}\right)}_{opt}$ and ${\left(\Delta E/k_{B}\right)}_{opt}$ obtained with four-objective optimization. In Figure 6a, the value of ${\left(E^{\prime }/k_{B}\right)}_{opt}$ ranges mainly from 12 to 13; as ${\left(E^{\prime }/k_{B}\right)}_{opt}$ grows, $\bar{R}$ continues to decline, ε continues to grow, $\bar{ECO}$ and $\bar{\chi }$ grow first and then decline. From Figure 6b, the value of ${\left(\Delta E/k_{B}\right)}_{opt}$ ranges mainly from 1.5 to 2.5; as ${\left(\Delta E/k_{B}\right)}_{opt}$ grows, $\bar{R}$ continues to grow, ε continues to decline, $\bar{ECO}$ and $\bar{\chi }$ grow first and then decline. From Figure 6, the values of $E^{\prime }$ and ΔE are closely related to the values of the four optimization objectives ($\bar{ECO}$, $\bar{R}$, ε and $\bar{\chi }$), and the selection of the parameters of the energy filter is very important to improve the performance of ESERs.

**Figure 7:**
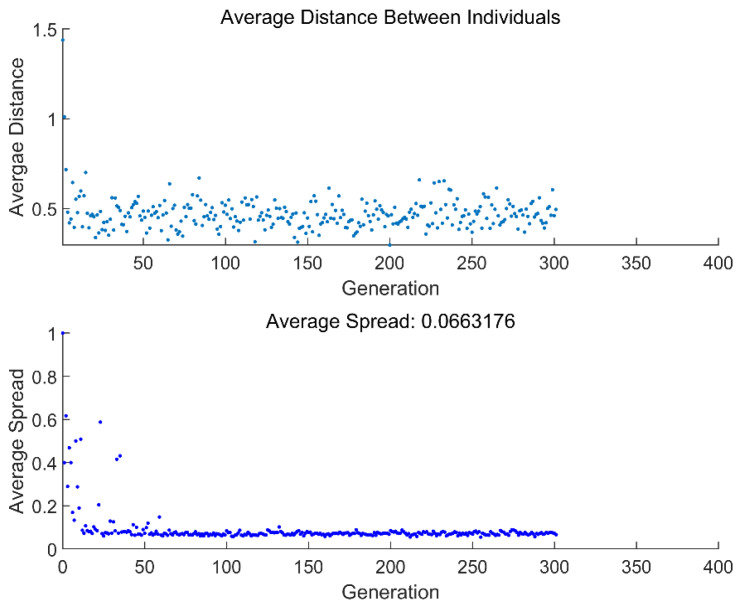
Average distance and average spread in relation to generations ($\bar{ECO}-\bar{R}-\varepsilon -\bar{\chi }$).

**Figure 8:**
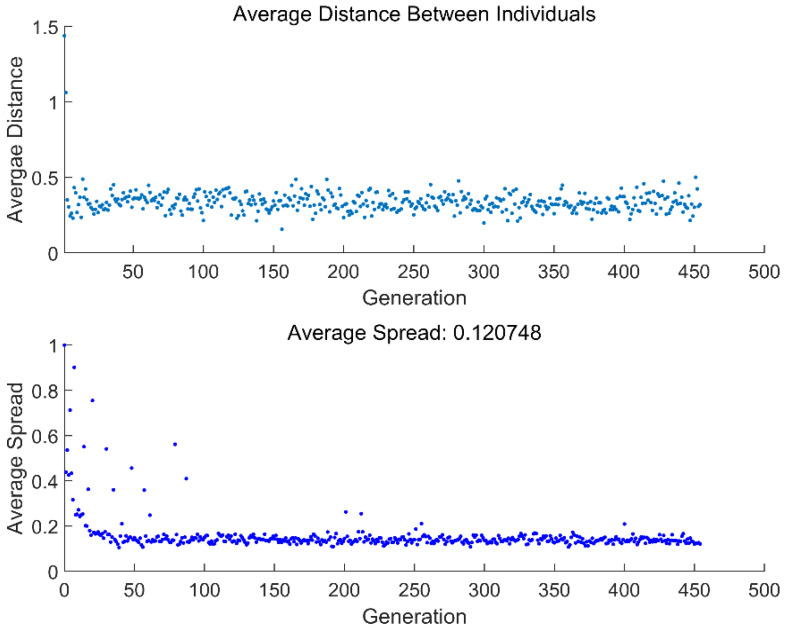
Average distance and average spread in relation to generations ($\bar{ECO}-\bar{R}$).

Figure 7 and Figure 8 give the average distance and average spread in relation to generations obtained from two different MOOs. From the two figures, the genetic algorithm will end when it reaches convergence, which occurs at the 302th and 455th generations for four-objective ($\bar{ECO}-\bar{R}-\varepsilon -\bar{\chi }$) and two-objective ($\bar{ECO}-\bar{R}$) optimizations, respectively.

## 4. Conclusions

In this paper, according to the model established in Ref. [89], the NSGA-II is applied to perform MOO for a single resonance energy selective electron refrigerator with heat leakage. Four objective functions are introduced, including cooling load, coefficient of performance, figure of merit, and ecological function. The $E^{\prime }$ and ΔE are regarded as optimization variables, their optimal intervals are obtained, and their effects on four objective functions are analyzed when the MOO is performed for $\bar{ECO}-\bar{R}-\varepsilon -\bar{\chi }$. The results show that:

1. The Ds obtained with LINMAP and TOPSIS approaches are 0.0812 for the MOO of $\bar{ECO}-\bar{R}-\varepsilon -\bar{\chi }$, which are more reasonable than those obtained with the SE approach; at this time, the values of $E^{\prime }/k_{B}$ and $\Delta E/k_{B}$ are 12.5958 and 1.8267, respectively. Comparing with the Ds (0.1085, 0.8455, 0.1865, and 0.1780) for the four single-objective optimizations with maximum $\bar{ECO}$, $\bar{R}$, ε, and $\bar{\chi }$, the Ds of the MOO are smaller. Therefore, compared with single-objective optimization, MOO can better take different optimization objectives into account by choosing appropriate decision-making methods.
2. When MOO is performed on $\bar{ECO}-\bar{R}$, the D is the 0.0809 obtained with the TOPSIS approach, which is the closest point to the positive ideal point and the most reasonable solution; and the corresponding values of the $E^{\prime }/k_{B}$ and $\Delta E/k_{B}$ are 12.5887 and 1.8050, respectively. When MOO is performed on other optimization objective combinations, the better solutions are obtained by choosing the appropriate decision-making approaches according to the design requirements.
3. For the MOO of $\bar{ECO}-\bar{R}-\varepsilon -\bar{\chi }$, the value of ${\left(E^{\prime }/k_{B}\right)}_{opt}$ ranges mainly from 12 to 13; as ${\left(E^{\prime }/k_{B}\right)}_{opt}$ grows, $\bar{R}$ continues to decline, ε continues to grow, $\bar{ECO}$ and $\bar{\chi }$ grow first and then decline. The value of ${\left(\Delta E/k_{B}\right)}_{opt}$ ranges mainly from 1.5 to 2.5; as ${\left(\Delta E/k_{B}\right)}_{opt}$ grows, $\bar{R}$ continues to grow, ε continues to decline, $\bar{ECO}$ and $\bar{\chi }$ grow first and then decline. It indicates that the values of $E^{\prime }$ and ΔE are closely related to values of the four optimization objectives ($\bar{ECO}$, $\bar{R}$, ε, and $\bar{\chi }$), and the selection of the parameters of the energy filter is very important to improve the performance of energy selective electron refrigerators.
4. For the MOO of $\bar{ECO}-\bar{R}-\varepsilon -\bar{\chi }$ and $\bar{ECO}-\bar{R}$, the average distances range mainly from 0 to 0.5 and change slightly; the average spreads range mainly from 0 to 0.2, vary significantly before the 100th generations, and then remain stable.
5. NSGA-II and FTT theory are effective tools to guide the designs of energy selective electron refrigerators.

## Figures and Tables

**Table 1:** Parameters of NSGA-II.

Parameters	Value
Generations	500
Population size	300
Pareto fraction	0.5
Crossover fraction	0.8

**Table 2:** Outcomes of single-, bi-, tri-, and quadru-objective optimizations.

Optimization Methods	Decision- Making Approaches	Optimization Variables		Objective Functions				Deviation Index
		$E^{\prime }/k_{B}$	$\Delta E/k_{B}$	$\bar{ECO}$	$\bar{R}$	ε	$\bar{\chi }$	*D*
Quadru-objective optimization ($\bar{ECO}$, $\bar{R}$, ε, and $\bar{\chi }$)	LINMAP	12.5958	1.8267	0.9036	0.7653	0.4467	0.9585	0.0812
	TOPSIS	12.5958	1.8267	0.9036	0.7653	0.4467	0.9585	0.0812
	SE	12.4042	1.9958	0.4687	0.8824	0.4042	1.0000	0.1780
Tri-objective optimization ($\bar{ECO}$, $\bar{R}$, and ε)	LINMAP	12.5761	1.8305	0.8771	0.7787	0.4428	0.9669	0.0814
	TOPSIS	12.5761	1.8305	0.8771	0.7787	0.4428	0.9669	0.0814
	SE	12.9850	1.4149	0.8242	0.4725	0.4916	0.6512	0.1873
Tri-objective optimization ($\bar{ECO}$, $\bar{R}$, and $\bar{\chi }$)	LINMAP	12.5699	1.8127	0.8673	0.7828	0.4415	0.9690	0.0821
	TOPSIS	12.5792	1.7916	0.8802	0.7763	0.4433	0.9650	0.0816
	SE	12.4042	1.9960	0.4687	0.8824	0.4042	1.0000	0.1780
Tri-objective optimization ($\bar{ECO}$, ε, and $\bar{\chi }$)	LINMAP	12.6624	1.7435	0.9702	0.7191	0.4592	0.9260	0.0888
	TOPSIS	12.6721	1.7141	0.9763	0.7121	0.4609	0.9202	0.0907
	SE	12.4042	1.9958	0.4687	0.8824	0.4042	1.0000	0.1780
Tri-objective optimization ($\bar{R}$, ε, and $\bar{\chi }$)	LINMAP	12.4130	1.9870	0.4975	0.8777	0.4063	0.9999	0.1692
	TOPSIS	12.3953	2.0053	0.4385	0.8871	0.4020	0.9999	0.1873
	SE	12.4041	1.9958	0.4685	0.8824	0.4042	1.0000	0.1781
Bi-objective optimization ($\bar{ECO}$ and $\bar{R}$)	LINMAP	12.5784	1.8420	0.8798	0.7771	0.4432	0.9657	0.0815
	TOPSIS	12.5887	1.8050	0.8951	0.7703	0.4454	0.9619	0.0809
	SE	11.9999	2.4007	−2.0171	1.0000	0.2943	0.8251	0.8455
Bi-objective optimization ($\bar{ECO}$ and ε)	LINMAP	12.8038	1.5968	0.9897	0.6141	0.4802	0.8268	0.1229
	TOPSIS	12.8038	1.5968	0.9897	0.6141	0.4802	0.8268	0.1229
	SE	12.9831	1.4169	0.8267	0.4739	0.4916	0.6533	0.1865
Bi-objective optimization ($\bar{ECO}$ and $\bar{\chi }$)	LINMAP	12.6601	1.7476	0.9686	0.7207	0.4588	0.9272	0.0884
	TOPSIS	12.6601	1.7476	0.9686	0.7207	0.4588	0.9272	0.0884
	SE	12.4042	1.9959	0.4686	0.8824	0.4042	1.0000	0.1780
Bi-objective optimization ($\bar{R}$ and ε)	LINMAP	12.4147	1.9849	0.5030	0.8768	0.4067	0.9999	0.5030
	TOPSIS	12.3785	2.0215	0.3786	0.8957	0.3979	0.9993	0.3786
	SE	12.9831	1.4168	0.8267	0.4739	0.4916	0.6532	0.8267
Bi-objective optimization ($\bar{R}$ and $\bar{\chi }$)	LINMAP	12.2286	2.1564	−0.3165	0.9594	0.3589	0.9655	0.4243
	TOPSIS	12.2391	2.1507	−0.2578	0.9559	0.3617	0.9695	0.4059
	SE	12.4042	1.9958	0.4686	0.8824	0.4042	1.0000	0.1780
Bi-objective optimization (ε and $\bar{\chi }$)	LINMAP	12.6184	1.8009	0.9307	0.7499	0.4511	0.9485	0.0824
	TOPSIS	12.6038	1.7766	0.9138	0.7599	0.4483	0.9552	0.0814
	SE	12.4041	1.9959	0.4685	0.8824	0.4042	1.0000	0.1781
Maximum $\bar{ECO}$	-	12.7500	1.6500	1.0000	0.6549	0.4732	0.8690	0.1085
Maximum $\bar{R}$	-	12.0000	2.4000	−2.0171	1.0000	0.2943	0.8252	0.8455
Maximum ε	-	12.9832	1.4168	0.8267	0.4739	0.4916	0.6532	0.1865
Maximum $\bar{\chi }$	-	12.4042	1.9958	0.4687	0.8824	0.4042	1.0000	0.1780
Positive ideal point		-	-	1.0000	1.0000	0.4916	1.0000	-
Negative ideal point		-	-	−2.0171	0.4739	0.2943	0.6532	-

## Nomenclature

A	Exergy output rate (W)
$E^{\prime }$	Energy boundary (J)
ECO	Ecological function
$eV_{0}$	Bias voltage
f	Fermi distributions of electrons
g	A defined function
h	Plank constant (J⋅s)
$k_{B}$	Boltzmann constant (J/K)
$k_{L}$	Heat leakage coefficient (W/K)
Q	Heat transfer (W)
R	Cooling load (W)
**Greek symbols**	
χ	Figure of merit (W)
ε	Coefficient of performance
μ	Electrochemical potential (J)
σ	Entropy generation rate (W/K)
ΔE	Resonance width (J)
**Subscripts**	
C	Cold reservoir
CE	Heat release rate
H	Hot reservoir
HE	Heat absorption rate
L	Heat leakage
opt	Optimal
0	Environment
**Superscripts**	
−	Dimensionless
**Abbreviations**	
CL	Cooling load
COP	Coefficient of performance
ESER	Energy selective electron refrigerator
FOM	Figure of merit
FTT	Finite time thermodynamics
MOO	Multi-objective optimization
SE	Shannon Entropy
